# Supplementary material for: Flow-induced crystallisation of polymers from aqueous solution
Source: Nat Commun. 2020 Jul 6;11:3372. doi: 10.1038/s41467-020-17167-8 (PMC7338548; doi:10.1038/s41467-020-17167-8)
Supplement: Supplementary file 1 — Supplementary Information [file 41467_2020_17167_MOESM1_ESM.pdf]

Supplementary Information

Flow-Induced Crystallisation of Polymers from Aqueous Solution

Dunderdale et al

Supplementary Table 1: PEO relaxation times.

Relaxation times (Rouse time of an entangled polymer segment, τ_e, disengagement time, τ_d, and Rouse relaxation time, τ_R) and a critical shear rate for polymer stretching, $\dot{\gamma}_R = \tau_R^{-1}$, for different molecular weights of PEO molecules, M (corresponding to both weight-average molecular weight M_w and higher-weight-average molecular weight M_z of PEO molecules used in this study, **Supplementary Fig. 4**), calculated at the chosen experimental temperatures, T, and some of their corresponding values (τ_{RC} and $\dot{\gamma}_{RC} = \tau_{RC}^{-1}$) corrected for a polymer mass concentration in the studied aqueous solutions, ϕ.

M, kDa	T, °C	τ_e, s	τ_d, s	τ_R, s	$\dot{\gamma}_R$, s^{-1}	ϕ	τ_{RC}, s	$\dot{\gamma}_{RC}$, s^{-1}
21.5	0	1.39×10^{-6}	1.62×10^{-3}	1.61×10^{-4}	6.23×10^{3}	0.5	1.42×10^{-5}	7.04×10^{4}
21.5	25	3.07×10^{-7}	3.58×10^{-4}	3.55×10^{-5}	2.82×10^{4}	0.6	5.94×10^{-6}	1.68×10^{5}
21.5	64	6.08×10^{-8}	7.09×10^{-5}	7.03×10^{-6}	1.42×10^{5}	1.0	7.03×10^{-6}	1.42×10^{5}
23.6	0	1.39×10^{-6}	2.27×10^{-3}	1.94×10^{-4}	6.23×10^{3}	0.5	1.71×10^{-5}	5.85×10^{4}
23.6	25	3.07×10^{-7}	5.01×10^{-4}	4.27×10^{-5}	2.82×10^{4}	0.6	7.15×10^{-6}	1.40×10^{5}
23.6	64	6.08×10^{-8}	9.93×10^{-5}	8.47×10^{-6}	1.42×10^{5}	1.0	8.47×10^{-6}	1.18×10^{5}
1799	0	1.39×10^{-6}	2.71×10^{3}	1.12	8.89×10^{-1}	0.5	9.94×10^{-2}	1.01×10^{1}
1799	25	3.07×10^{-7}	5.98×10^{2}	2.48×10^{-1}	4.03	0.6	4.16×10^{-2}	2.41×10^{1}
1799	64	6.08×10^{-8}	1.18×10^{2}	4.92×10^{-2}	2.03×10^{1}	1.0	4.92×10^{-2}	2.03×10^{1}
2851	0	1.39×10^{-6}	1.10×10^{4}	2.82	3.54×10^{-1}	0.5	2.50×10^{-1}	4.01
2851	25	3.07×10^{-7}	2.44×10^{3}	6.24×10^{-1}	1.60	0.6	1.04×10^{-1}	9.58
2851	64	6.08×10^{-8}	4.83×10^{2}	1.24×10^{-1}	8.09	1.0	1.23×10^{-1}	8.09

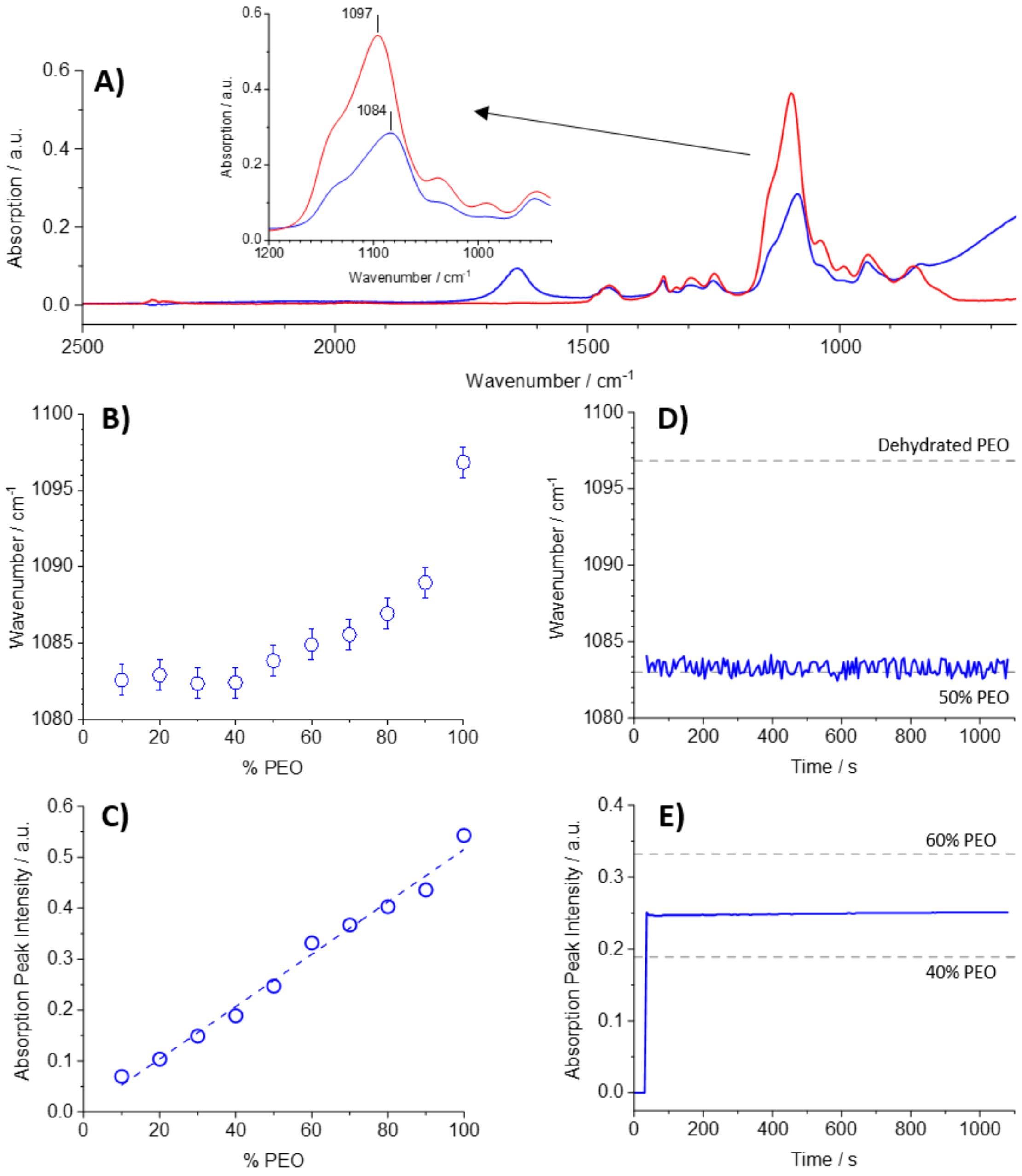

Supplementary Fig. 1: FTIR spectroscopy of PEO at 80 °C.

FTIR spectroscopy measurements were performed using ATR attachment mounted on a rheometer (**Methods**). The samples were maintained at 80 °C in a humidity control environment similar to the SIPLI setup (**Methods**). **A)** Infrared absorption **s**pectra of a PEO melt (100% w/w) (red line) and 50% w/w PEO aqueous solution (blue line) at 80 °C. Inset shows a magnified view of the PEO ether (C-O-C) stretching band peak which changes its frequency and intensity depending on the hydration state and concentration of PEO, respectively. The concentration dependence of the C-O-C stretching band peak at 1080-1100 cm^{-1} is shown in **B)** peak frequency and **C)** peak intensity. For the PEO melt (completely dehydrated PEO) the peak position is about 1097 cm^{-1} and peak intensity greatest. As PEO becomes more hydrated the peak position moves to lower wavenumber, **B)**, eventually settling at around 1083 cm^{-1} when no more water molecules can be accommodated by the PEO hydration shell, and the peak intensity reduces demonstrating a linear dependence on PEO concentration, **C)**. Monitoring of the solvation state and composition of 50% w/w PEO aqueous solution by measuring position and intensity of the ether stretching band peak, **D)** and **E)**, respectively. As 50% w/w PEO aqueous solution is added

to the apparatus at Time = 30 s, the intensity jumps from zero to a value which is constant over time, indicating that evaporation of water is insignificant and that the composition remains at 50% w/w PEO, **E)**. For a comparison, dashed lines indicate the intensities measured at 40% w/w and 60% w/w PEO concentrations. The wavenumber of the C-O-C stretching band peak also maintains a constant value, highlighting that the solvation state of PEO remains constant, **D)**. No phase separation or crystallization occurs as would be indicated by a migration towards dehydrated PEO having a peak wavenumber of 1097 cm^{-1} as shown by the dashed line. For a reference, the interval of this experiment is nearly 8 times longer than the total heat treatment period of 120 s used for a sample homogenization at elevated temperatures (80 °C) before performing SIPLI measurements. Error bars indicate uncertainty of measurements.

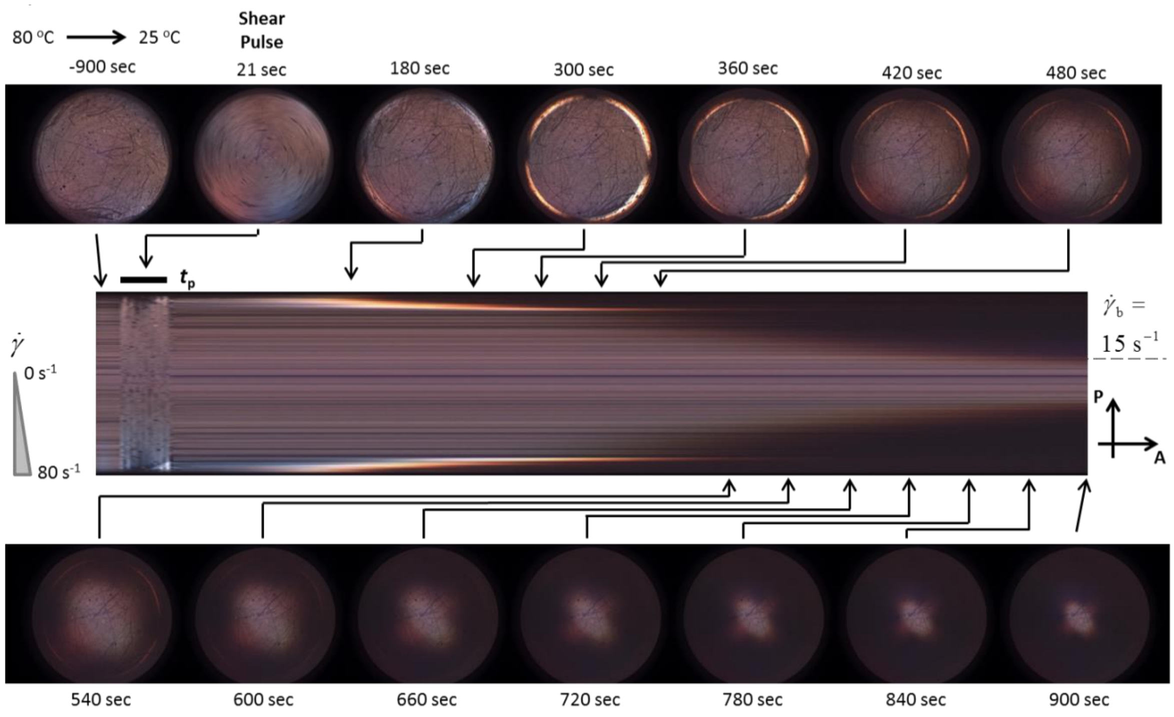

Supplementary Fig. 2: Measurement of conditions required for flow-induced nucleation of PEO in water. Representative polarised light images (circular images) of a sheared 60% w/w PEO aqueous solution recorded during a SIPLI experiment (R = 12.5 mm, d = 0.5 mm, ω = 3.2 rad/s and t_p = 51 s), with a time-lapse (rectangular image) composed of 45° slices through images over the course of the measurement (**Methods**). The vectors assigned by P and A show polarizer and analyzer axis, respectively.

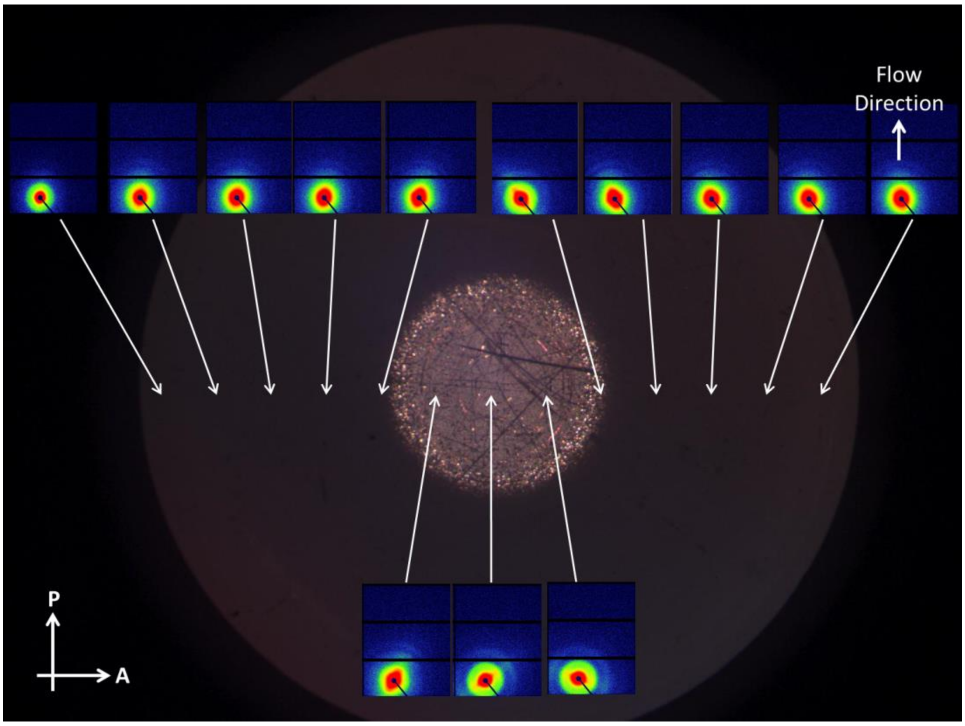

Supplementary Fig. 3: Flow-induced crystallization of the PEO bimodal blend.
SIPLI image of a sheared bulk PEO bimodal blend (R = 12.5 mm, d = 0.5 mm, ω = 1.2 rad/s and t_p = 33 s, at a temperature of 64 °C), then cooled to 62 °C to promote crystallization. Insets are SAXS patterns captured across the sample at the positions indicated by the arrows after the sample had been cooled to room temperature and removed from the SIPLI rheometer. The vectors assigned by P and A show polarizer and analyzer axis, respectively.

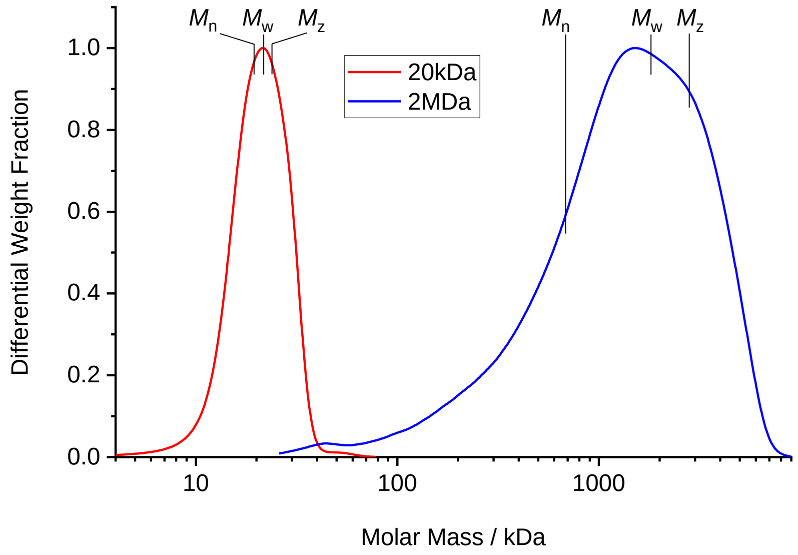

Supplementary Fig. 4: Molar mass distribution of PEO polymers used in this study.

Size exclusion chromatograms of PEO with nominal molecular weight of 20kDa (red line) and 2 MDa (blue line). Corresponding number-average molecular weight, M_n, weight-average molecular weight, M_w, and higher-weight-average molecular weight, M_z, of the polymers (19.3 kDa, 21.5 kDa and 23.6 kDa for PEO with nominal molecular weight of 20kDa and 678 kDa, 1799 kDa and 2851 kDa for PEO with nominal molecular weight of 2 MDa, respectively) are indicated on the plot.

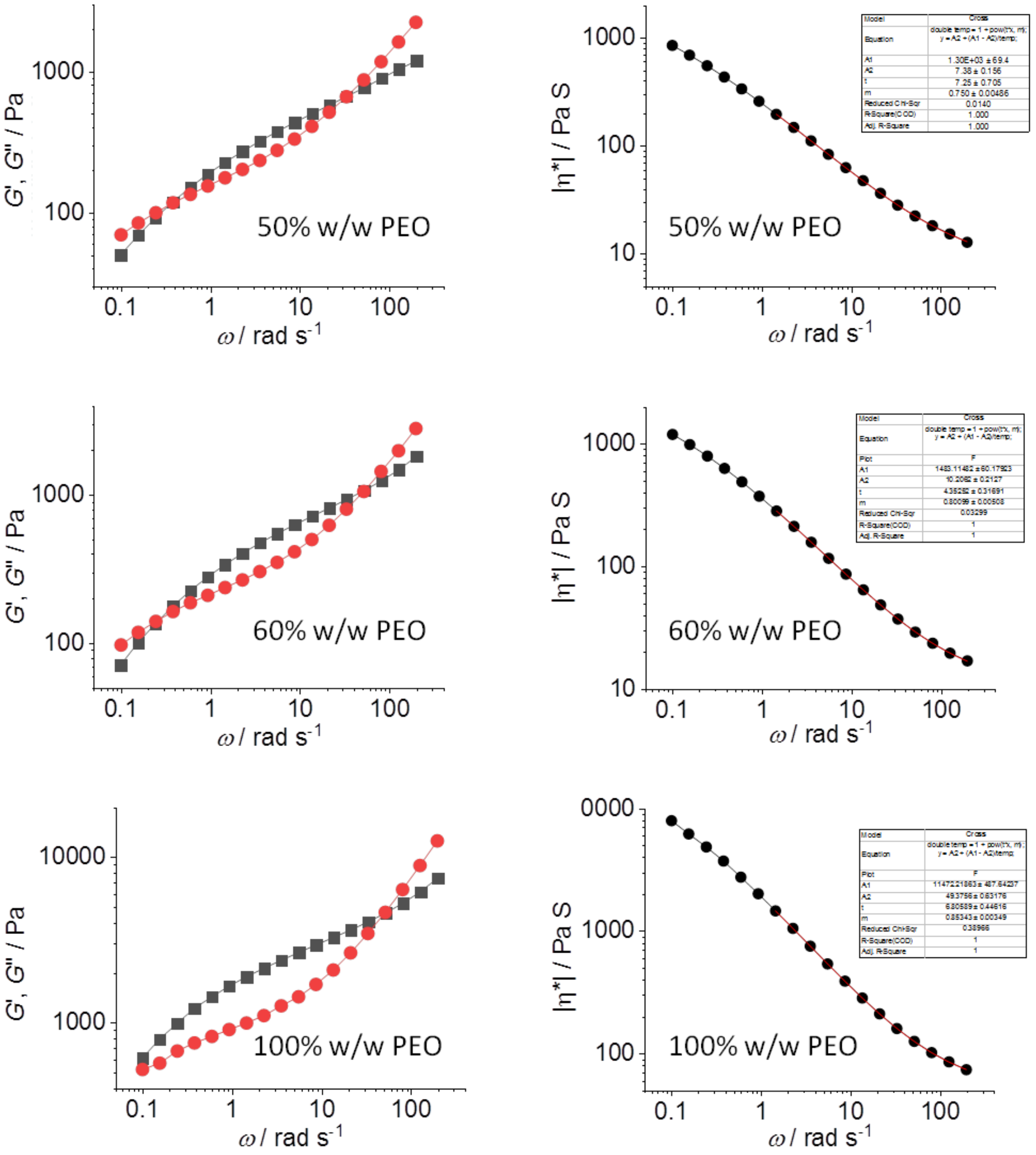

Supplementary Fig. 5: Rheological properties of PEO aqueous solutions.
Left column of the graphs – frequency sweeps of 50, 60, and 100% w/w PEO solutions at 0 °C, 25 °C, and 64 °C, respectively. *G*' (red symbols) and *G*'' (black symbols) is storage and loss modulus, respectively. Right column of the graphs – modulus of complex viscosity calculated from the frequency sweeps, and fitted with a Cross Model (red line). Cross model fitting parameters (**see Methods**) are inserted in each graph as a table.

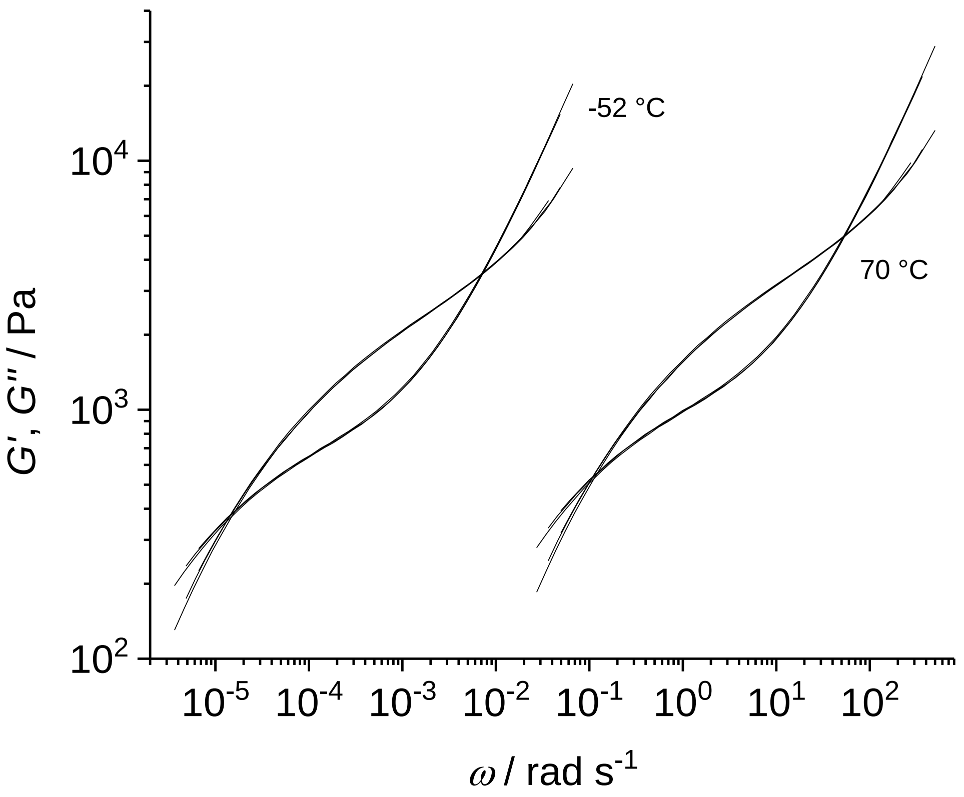

Supplementary Fig. 6: Rheological master curves for PEO bimodal blend.
Storage (*G*') and loss (*G*'') moduli angular frequency dependence of studied 2 MDa in 20 kDa PEO bimodal blend at glass transition temperature (T_{ref} = -52 °C) and T_{ref}^{new} = 70 °C. The experimental data were collected at 70 °C, 80 °C and 90 °C and then initially shifted to PEO glass transition temperature (T_{ref} = -52 °C) using horizontal shift coefficient (a_T, **see Methods**) and vertical shift coefficient (b_T, **see Methods**).

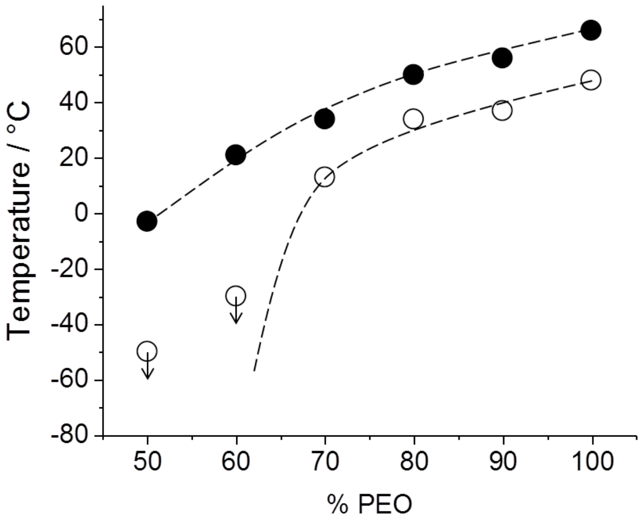

Supplementary Fig. 7: Melting and crystallization temperature of PEO.
Melting (filled points) and crystallization temperatures (hollow points) measured by DSC plotted against composition. Crystallization points with an arrow indicate that crystallization was not observed as the sample was cooled, but was the crystallisation point on subsequent heating of the supercooled liquid, and as such these data indicate temperatures below which the crystallization must occur.

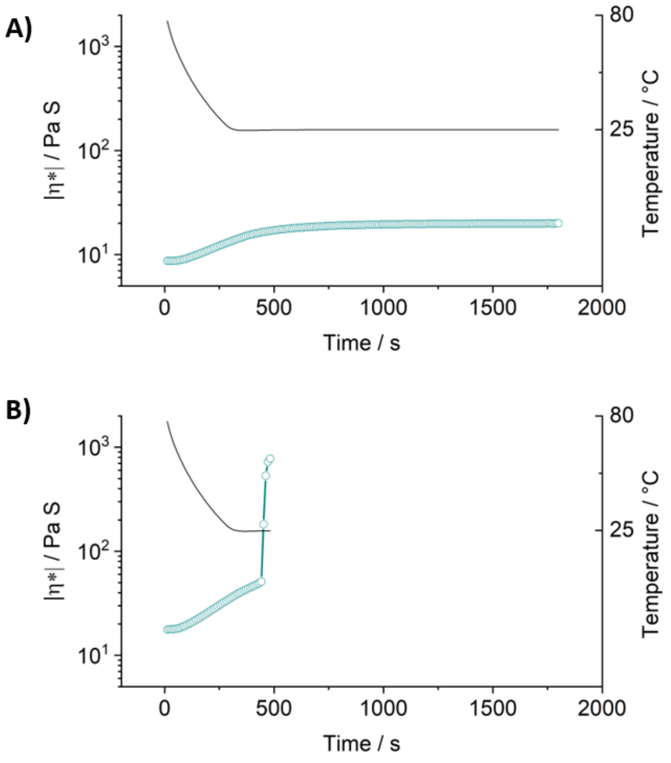

Supplementary Fig. 8: Monitoring of PEO dehydration by rheology.
A 60% w/w PEO aqueous solution is heated to 80 °C, then at Time = 0 s cooled down to processing temperature (25 °C) over ~ 250 s (black line). The magnitude of complex viscosity is measured over time using a rheometer (oscillatory mode, angular frequency 10 rad/s at 0.1% strain) with and without solvent trap, **A)** and **B)**, respectively. If the solvent trap is used, **A)**, this results in a sample environment with a relative humidity of ~ 97% as measured by a humidity probe. Under these conditions the magnitude of complex viscosity increases as the sample cools eventually reaching an equilibrium value, evaporation does not occur to any significant extent over the shear experiment timescale used for the sample equilibration (900 s) and subsequent shearing (0-100 s) to trigger flow-induced nucleation of PEO. However, if the solvent trap is not used, **B)**, water is lost through evaporation during the heating resulting in a slightly higher initial viscosity (Time = 0 s) which continues to increase as further water is lost during the sample cooling. Eventually, the composition of the sample at the edge of the rheometer plate becomes high enough in PEO and solidification occurs at the reduced temperature. This results in a sharp increase in the measured viscosity by almost two orders of magnitude. That is to say, as the PEO hydration shell becomes incomplete due to evaporation, and the temperature decreases, the PEO aqueous solution is unstable and at some point PEO crystallises resulting in a significant increase of the magnitude of complex viscosity. This comparison shows that using the solvent trap for rheological experiments prevents PEO dehydration by evaporation.
